# Service years of health professionals are associated with tuberculosis infection control practice in Ethiopian Teaching Hospital

**DOI:** 10.11604/pamj.2021.38.253.23044

**Published:** 2021-03-11

**Authors:** Wondimagegn Wondimu, Tewodros Yosef, Tadesse Gebremedhin, Mohammed Muze

**Affiliations:** 1 Mizan-Tepi University, College of Medicine and Health Sciences, School of Public Health, Department of Epidemiology and Biostatistics, Mizan Aman, Ethiopia,; 2 Jimma University, Faculty of Public Health, Department of Epidemiology, Jimma, Ethiopia,; 3 Bench Sheko Zone Health Department, Mizan Aman, Ethiopia

**Keywords:** Tuberculosis infection control practice, practice, health professionals, Mizan Tepi University Teaching Hospital

## Abstract

**Introduction:**

proper tuberculosis (TB) infection control (TBIC) practice of health professionals is one of the effective TB prevention approaches. Despite this reality, the TBIC practice of health care workers was not been well studied. This study assessed the TBIC practice of health professionals and associated factors in Mizan Tepi University Teaching Hospital, southwest Ethiopia.

**Methods:**

an institution based quantitative cross-sectional study was conducted from September 1 to 30, 2019, by including all health professionals in the hospital. Participants who answered at least 50% of TBIC practice questions correctly were categorized as having good TBIC practice. Binary logistic regression was used to identify factors associated with the practice of the participants. The odds ratio with a 95% confidence interval and p-value was used to measure the strength of association; the significant association was declared at a p-value less than 0.05.

**Results:**

the study found that 64.1% (95% CI: 56.6%, 70.7%) of the participants had good TBIC practice. More than half, 102(51.5 %) of study participants have service years of greater than or equal to five years. Only the service year of health professionals was significantly associated [95%CI (AOR= 2.43; 95%CI: 1.28, 4.6)] with the respondents´ TBIC practice.

**Conclusion:**

only less than two-third of health professionals had good TBIC practice which is inadequate. And also experienced staff had significantly better TBIC practice. As a result, health professionals in MTUTH should be supported to practice TBIC as routine activity and opportunities should be made for senior staffs to share their TBIC experiences with others.

## Introduction

Tuberculosis (TB) is a communicable disease and one of the major causes of morbidity and mortality [1]. Globally it is among the top ten causes of mortality and with this it ranks above HIV/AIDS. Moreover, it affects all people regardless of their sex and age groups [2]. TB can be easily transmitted from one person to another mostly when TB patients expel TB causing bacteria into the air through coughing [1]. The World Health Organization (WHO) estimate of 2020 shows that globally there were a total of 1.5 million deaths from TB in 2018 (including 251,000 people with HIV). In the same year, an estimated 10 million people (5.7 million men, 3.2 million women, and 1.1 million children) fell ill with TB worldwide. TB occurs in every part of the world and the largest number of new TB cases occurred in the South-East Asian region (44%), followed by the African region (24%). The highest proportion (87%) of the new TB cases occurred in 30 high TB burden countries. Ethiopia is among these 30 high TB burden countries [3].

To combat the high prevalence of TB morbidity and mortality, WHO announced an end TB strategy [4]. Therefore, to achieve this there should be effective TB diagnosis, treatment, control, and prevention approaches [2, 4]. Health care providers play great roles in the prevention and control of TB. However, they have the highest risk of exposure to TB infection [5, 6]. One of the effective TB prevention and control mechanisms for both health workers (HCWs) and clients in health institutions is the good practice of health care providers towards tuberculosis infection control (TBIC) measures [7]. TBIC measures consist of a combination of measures designed to minimize the risk of tuberculosis transmission within the health care settings and other environments. The WHO recommends that the TBIC program should be implemented based on the three levels of hierarchy. These include administrative controls (reduce risk of exposure), environmental controls (prevent the spread and reduce the concentration of droplet nuclei) and respiratory infection control (further reduce risk of exposure in special areas and circumstances) [4].

HCWs should be encouraged to practice TBIC measures [7, 8]. TBIC measures must be strengthened in line with the introduction of latent TB infection screening and treatment of HCWs [9]. Different studies revealed that despite a good knowledge and positive attitude towards TBIC, most of the health care workers in different settings were not practicing TBIC [7, 8]. Available studies also reported that TBIC knowledge, training on TBIC, working site, the age of respondents, and profession were significantly associated with self-reported TBIC practice of HCWs [5, 7, 8, 10-12]. However, there were limited studies towards the practice of TBIC in the country as a whole and there was no study done in the study setting. Therefore, this study assessed the self-reported practice of TBIC and associated factors among health professionals in Mizan Tepi University Teaching Hospital (MTUTH), southwest Ethiopia.

## Methods

### Study design, area and period

An institution based quantitative cross-sectional study was conducted in Mizan Tepi University Teaching Hospital (MTUTH) from September 1 to 30, 2019. MTUTH is located at 593 km to the southwest of Addis Ababa, the capital of Ethiopia. Majority of the clients in this hospital were from the Bench Sheko zone and partly from the Kaffa zone and Gambella regional state. Emergency case team, adult and pediatric outpatient departments´ case teams, maternal and child health service case teams, antiretroviral therapy case team, and obstetrics and gynecology case teams are among the different departments in the hospital. There was a separate TB clinic for TB patients and the hospital had 218 health professionals during the time of the study.

### Study population, data collection, management and analysis

All health professionals were included in the study. The health professionals who didn´t involve directly in serving TB patients or those who were serving in a manner that will not put them to TB contact were excluded. Data were collected by trained data collectors using a structured self-administered questionnaire which was adapted from the previous related study [8]. Questions that assess socio-demographic and other general characteristics, knowledge, attitude, and practice of TBIC were included in the questionnaire. Principal investigators supervised the overall data collection process through immediate and daily checkups of the questionnaire to avoid incompleteness and inconsistency. Epi-data manager version 4.0.2.101 and SPSS version 21 were used for data entry and analysis respectively. The practice of TBIC (categorized as good and poor) was the dependent variable. The explanatory variables include socio-demographic characteristics (age, sex, and educational level), current profession, job location, service year, TBIC training, knowledge of TBIC, and attitude towards TBIC.

Health professionals who scored at least 50% of practice questions correctly were categorized as practicing TBIC (had good TBIC practice) otherwise not practicing TBIC (had poor TBIC practice). The cutoff point was obtained from the previous local study [8]. The association between dependent and independent variables was assessed using the binary logistic regression model. The odds ratio with a 95% confidence interval (CI) and p-value was used to measure the strength of association. Variables with the p-value of less or equals to 0.25 in bi-variable logistic regression were considered as eligible for multivariable analysis. In multivariable analysis, independent variables with the p-value of less than 0.05 were declared as significantly associated with an outcome variable.

### Ethical consideration

The research proposal was approved by the ethical review committee of health science college, Mizan Tepi University, and ethical clearance was obtained. The support letter was written to the hospital from Mizan Tepi University. The purpose of the study was briefed to the respondents and consent was obtained. The study participants had the right to participate and not to participate. The name of the respondents was not collected and the identification number was assigned for each participant. The issue of confidentiality was ensured strictly and the collected data were kept in the locked cabinet until the final safe removal.

## Results

### Socio-demographic and other general characteristics of respondents

There were 215 health professionals eligible for this study and 198 fully participated, making the response rate of 92.1%. The majority, 109 (55.1%) of the respondents were females and of them, 75 (68.8%) had good TBIC practice. More than half of the participants had a history of TBIC training (52.5%); among whom 61.5% had a good practice of TBIC measures. About half (51.5%) of the respondents had at least 5 years´ experience and 72.5% of them had good TBIC practice (Table 1).

**Table 1:** sociodemographic and other general characteristics of respondents in MTUTH, 2019

Variable	Category	N	%	Practice			
				Poor		Good	
				n	%	n	%
**Age**	≤30	127	64.1	44	34.6	83	65.4
	31-40	63	31.8	23	36.5	40	63.5
	>40	8	4	4	50	4	50
**Sex**	Male	89	44.9	37	41.6	52	58.4
	Female	109	55.1	34	31.2	75	68.8
**Educational level**	Diploma	96	48.5	27	28.1	69	71.9
	First degree	98	49.5	42	42.9	56	57.1
	Second degree and above	4	2	2	50	2	50
**History of TBIC training**	Yes	104	52.5	40	38.5	64	61.5
	No	94	47.5	31	33	63	67
**Service year**	< 5 years	96	48.5	43	44.8	53	55.2
	≥5 years	102	51.5	28	27.5	74	72.5
**Job location**	OPD	44	22.2	11	25	33	73
	Ward	62	31.3	25	40.3	37	59.7
	Pharmacy/laboratory/other unit	92	46.5	35	38	57	62
**Current profession**	Physician/Public health	55	27.8	26	47.3	29	52.7
	Nurse/Midwifery	83	41.9	27	32.5	56	67.5
	Pharmacist /others	60	30.3	18	30	42	70

### Practice of TBIC measures and associated factors

Among 198 participants, about two-third of them (65.2%) always open the window whenever a suspected or confirmed TB case is in their room. Similarly, only 28.3% give priority to coughing patients in the waiting area (Table 2). Generally, 64.1% (95% CI: 56.6%, 70.7%) of the participants had good TBIC practice. The candidate variables for multivariable logistic regression include sex, current profession, job location, service year, knowledge of TBIC, and attitude towards TBIC. In the multivariable logistic regression, only the service year of the participants was significantly associated with the practice of TBIC (Table 3).

**Table 2:** health professionals´ practice of TBIC in MTUTH, 2019

Questions	Responses					
	Always		Sometimes		Never	
	N	%	N	%	N	%
Do you open the window whenever a patient suspected or confirmed to have TB is in the room?	129	65.2	66	33.3	3	1.5
Do you use a mask/respirator whenever you are treating TB patients/suspects?	120	60.6	71	35.9	7	3.5
Do you try to see coughing patients at first, in other words, if there are coughing patients in the waiting area, do you give them priority?	56	28.3	132	66.7	10	5.1
Do you educate your TB patients on cough etiquette? (That is covering of mouth while coughing, not spitting on the floor, etc)	157	79.3	30	15.2	11	5.6
If you were supplied with fans, would you turn them on while you are treating TB suspects or confirmed cases?	106	53.5	84	42.4	8	4.0
Having been in contact with TB patients, would you test for TB in case you have a cough?	57	28.8	130	65.7	11	5.6

**Table 3:** bi-variable and multivariable logistic regression of factors associated with the practice of TBICs among health professionals in MTUTH, 2019

Variable	Category	COR	P-value	AOR (95% CI)	P-value
**Sex**	Male	0.64	0.131	0.64 (0.34, 1.19)	0.157
	Female	1		1	
**Current profession**	Physician/ Public health	0.06	0.478	0.57 (0.24, 1.36)	0.206
	Nurse/Midwifery	0.75	0.889	0.76 (0.34, 1.72)	0.511
	Pharmacist /others	1		1	
**Job location**	OPD	1.84	0.135	2.08 (0.88, 4.88)	0.095
	Wards	0.91	0.776	1 (0.47, 2.12)	0.996
	Other case teams	1		1	
**Service year**	< 5 years	1		1	
	≥5 years	2.14	0.012	2.43 (1.28, 4.6)	0.007*
**Knowledge of TBIC**	Poor	1		1	
	Good	0.64	0.18	0.89 (0.42, 1.91)	0.767
**Attitude toward TBIC**	Negative	1		1	
	Positive	0.63	0.221	0.78 (0.34, 1.79)	0.552

* Statistically significant at p-value less than 0.05

## Discussion

The roles of health professionals in preventing TB are crucial. These roles are highly predicted by their practice towards TBIC in health care settings. Our study assessed the practice of TBIC and associated factors among health professionals in MTUTH. In this study; 64.1% (95% CI: 56.6%, 70.7%) of health professionals had a good practice of TBIC. This finding is almost similar to that of a study conducted in Northwest Ethiopia which revealed that 63.3% of health professionals had good practice [8]. In contrast to this, the study done in Gondar and Felege Hiwot Referral Hospitals, Ethiopia, reported that 19.6% of health professionals had good practice towards TBIC [10]. This discrepancy could be due to the variations in sample size and the difference in the data collection tool. An additional explanation might be that the current study focused on general TBIC but the study done in Gondar and Felege Hiwot Referral Hospitals was mainly on MDRTB [10].

The current study showed that there is no significant difference between male and female respondents with TBIC practice (AOR=0.64; 95% CI: 0.34, 1.19). This is also in line with the study conducted in Debremarkos and Bahirdar settings. In addition to this, the current study is also comparable with the study done in Gondar University and Felege Hiwot Referral Hospitals which revealed that there was no significant difference in TBIC practice among male and female health professionals [8, 10]. This study showed that 79.3% (95%CI: 73.2, 84.8%) of health professionals gave education to the patients regarding the proper way of coughing to prevent TB infection transmission. This is nearly supported by the study conducted in Northwest Ethiopia which revealed that 71% of study participants gave education on how to prevent TB infection transmission [8]. However, the current finding is higher compared to that of another Ethiopian study [12]. Our study showed that health professionals who had at least five years service in the hospital had a good practice of TBIC than their counterparts (AOR=2.43; 95% CI: 1.28, 4.6). This could be due to the experiences they got from different senior staffs and trainings they attended throughout their long stay in the job.

### Limitation of the study

This study tried to include all health professionals to eliminate sampling error and non-representativeness. In spite of this strength, since we did not use observation as a data collection method there might be an overestimation in the prevalence of good practice due to social desirability bias. For the future researchers, we would like to recommend different data collection approaches such as observation to reduce the probability of getting socially acceptable responses.

## Conclusion

Although almost all of the health professionals in MTUTH have the probability of contacting TB patients, only less than two-third of them had good TBIC practice and the service year was significantly associated with good practice of TBIC. As a result, health professionals in MTUTH should be supported to practice TBIC as routine activity and opportunities should be made for senior staffs to share their TBIC experience with others.

### What is known about this topic

- In spite of good knowledge and positive attitude towards TBIC, most of the health care workers in different settings were not practicing TBIC;
- TBIC knowledge, training on TBIC, working site, the age of respondents, and profession were significantly associated with self-reported TBIC practice of HCWs.

### What this study adds

- In contrary to the expectation, only less than two-thirds of the participants were practicing TBIC measures properly;
- The service year of health professionals (which was not reported in previous works) is significantly associated with a good TBIC practice;
- Unlike previous findings, the knowledge and attitude of TBIC were not significant determinants.
